# Origin of Canine Distemper Virus: Consolidating Evidence to Understand Potential Zoonoses

**DOI:** 10.3389/fmicb.2019.01982

**Published:** 2019-08-28

**Authors:** Carolina Quintero-Gil, Santiago Rendon-Marin, Marlen Martinez-Gutierrez, Julian Ruiz-Saenz

**Affiliations:** ^1^Grupo de Investigación en Ciencias Animales–GRICA, Universidad Cooperativa de Colombia, Bucaramanga, Colombia; ^2^Infettare, Facultad de Medicina, Universidad Cooperativa de Colombia, Medellín, Colombia; ^3^Asociación Colombiana de Virología, Bogotá, Colombia

**Keywords:** distemper, evolution, morbillivirus, measles, zoonotic disease

Morbilliviruses are highly contagious pathogens and are responsible for various outbreaks in unexposed populations (Pfeffermann et al., 2018). They belong to the order Mononegavirales and family *Paramyxoviridae* and are characterized by a non-segmented, linear, negative-stranded RNA genome (Lamb and Parks, 2013). Morbilliviruses are distinguished for causing moderate-to-severe respiratory, gastrointestinal, immunosuppression, and/or neurological diseases in a wide range of hosts, including humans (measles virus), carnivores (canine morbillivirus formerly canine distemper virus), cattle (rinderpest virus), dolphins and porpoises, and other wildlife-endangered species (Lamb and Parks, 2013; Martinez-Gutierrez and Ruiz-Saenz, 2016).

Measles virus (MeV) and canine morbillivirus (CDV) are considered the most contagious viruses among this family (De Vries et al., 2015), and due to the high transmission potential of CDV as well as its cross-species transmission potential, the global health, and conservationist authorities are greatly concerned about role of CDV on endangered species conservation and the possible “jump” from animals to humans (Terio and Craft, 2013; Ohishi et al., 2014). Domestic dogs are the main host for CDV and could also be considered as a reservoir for other mammals (Suzuki et al., 2015; Duque-valencia et al., 2019); however, based on the biology of CDV, humans could also turn into a potential target (Cosby and Weir, 2018; Rendon-Marin et al., 2019).

Trying to understand the potential risk of transmission of CDV to humans, it is necessary to gather all the existing evidence; and the study of the origin and dissemination of this agent in the canine population could present an important key to understanding this process. Recently, a paper published in the *International Journal of Paleopathology* invited to a discussion on the evolutionary origin of CDV. It concludes that CDV originated as a pandemic pathogen in South America following the infection and adaptation of MeV to dogs during the South American colonization period. This result was obtained via an interdisciplinary approach adopted by synthesizing a paleopathological analysis of 96 pre-Columbian dogs (750–1470 CE) from the Weyanoke Old Town, Virginia site, with historical reports, molecular analysis, and morbilliviral epidemiology (Uhl et al., 2019).

Notably, native dog populations from America almost disappeared after the colonization period, and European and Eurasian dogs were introduced to the continent, leaving little genetic background of its American predecessors (Ni Leathlobhair et al., 2018). Another important factor worth considering is that “unknown” diseases could have also been introduced, making it harder to track the origin of new pathogens. Moreover, artificial selection pressure over domestic dogs and even human populations, particularly during the colonization period, could have enhanced disease incidence, thereby limiting genetic variation (Ostrander et al., 2017), which in turn could mean less effective response against pathogens.

Among these “new” pathogens/diseases, CDV was first described by Antonio de Ulloa y de la Torre-Giral in 1746 as a disease affecting dogs in the Quito region and the other parts of South America, and it was reported soon afterward in Europe. CDV was recorded in Spain in 1760, with 900 deaths occurring in a single day in Madrid, and 3 years later, i.e., by 1764 and 1770, it had reached Great Britain and Italy, respectively (Blancou, 2004). Virus transmissibility and greater susceptibility of puppies compared with adult dogs were later reported by Edward Jenner in the early 1800s. He compared their transmissibility with that of MeV and discovered that survivors were protected from subsequent infection (Jenner, 1809; Nambulli et al., 2016).

Briefly, after the arrival of European pioneers in the fifteenth century, novel infectious diseases arguably became the most devastating consequence of colonization because the indigenous American populations had no prior exposure to pathogens that had become common in Europe (Walker et al., 2015). Multiple measles epidemics, therefore, devastated the indigenous American populations (Walker et al., 2015; Nambulli et al., 2016). Uhl et al. via a mixed approach of paleopathological, historical, molecular, and epidemiological evidence, reported that severe MeV epidemics in the indigenous American populations facilitated the jump of MeV to large domestic dog populations of urban environments in South America and the adaptation of the virus as endemic CDV (Uhl et al., 2019). Also, historical records could prove that few years after that adaptation to South American dogs, CDV was transported to Europe in 1760, where it initially induced widespread epidemics with high mortality before becoming endemic (Jenner, 1809).

However, molecular phylogeography related to evolutionary predictions and the time to the most recent common ancestor (tMRCA) were calculated for the CDV origin in the United States in the 1880s (95% highest posterior density, 1858–1913) (Panzera et al., 2015), which clearly contradicts the description of the virus in Europe in the eighteenth century. Sequence analyses that led to this hypothesis must be carefully examined because of the bias and the limited availability of sequences that were used in this molecular phylogeography reconstruction. Moreover, many original ancestral sequences have been lost due to the lability of the viral RNA genome of the CDV and other morbilliviruses. These factors have given rise to the questioning of the utility of current tMRCA calculations for RNA viruses (Sharp and Simmonds, 2011; Nambulli et al., 2016).

According to Uhl et al., morbillivirus could have originated from cattle around 376 BC in the “old continent” (Figure 1), and animal domestication may have had a significant influence on cross-species events, probably tracing a starting point in MeV emergence to approximately 900 AC (Uhl et al., 2019). Contrary to the current CDV phylogenetic reconstructions, MeV divergence is strongly supported by the relaxed clock Bayesian phylogenetic analysis. The divergence time between MeV and the rinderpest virus had been shown to have occurred in approximately the eleventh to twelfth centuries (Furuse et al., 2010). Other molecular data, such as the presence of a new morbillivirus (closely related to CDV and PDV) circulating in bats from Brazil (DrMV), allows the speculation that CDV and DrMV might share a common South American ancestor (Drexler et al., 2012), thereby indirectly supporting the idea of the early South American Origin of CDV.

**Figure 1:**
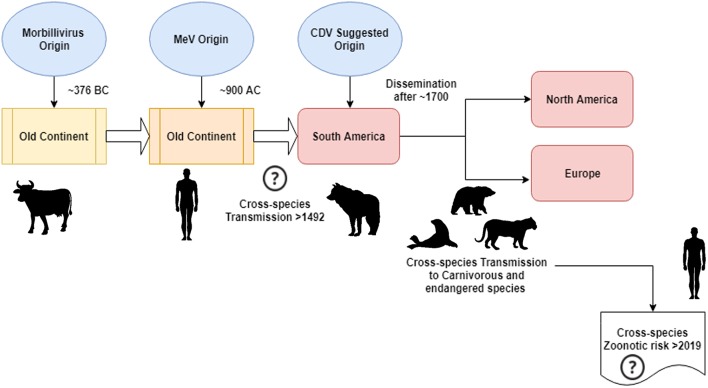
Schematic representation of the possible canine morbillivirus (CDV) evolutionary transmission route. See text for references.

Beyond the epistemological and/or scientific meaning of the geographical origin and date of CDV divergence, there are important clues that must be clarified to better understand the current impact of CDVs on interspecies transmission, animal conservation, and zoonotic potential (Figure 1). It is clear that unlike the MeV infection, which is maintained by a single host (humans), CDV has been widely proved to be a promiscuous pathogen-causing infection/disease in a vast array of carnivorous and non-carnivorous species (Martinez-Gutierrez and Ruiz-Saenz, 2016). This promiscuity has been attributed to not only the capacity of the CDV hemagglutinin (H) to interact with host cellular receptors, such as SLAM in mononuclear cells and nectin-4 in epithelial cells, but also the similarity among species sequences of the receptors mentioned above (Rendon-Marin et al., 2019). The amino acid similarity among mammal SLAM receptors, including marine mammals, is >80% (Ohishi et al., 2014), thereby supporting the results of cross-species transmission. In addition, there is a lack of species-related variation in the nectin-4 sequences among humans, mice, and dogs because human nectin-4 could function as an *in vitro* receptor for CDV (Noyce et al., 2011).

Natural CDV outbreaks in different non-human primates have raised a concern regarding the possible transmission of CDV to humans (Yoshikawa et al., 1989; Sun et al., 2010; Qiu et al., 2011; Sakai et al., 2013a). There are reports that CDV monkey strains have the intrinsic ability to use human nectin-4 for virus entry and that those monkey CDVs easily adapt to use the human CD150 (SLAM) receptor following minimal amino acid changes to the viral H protein (Bieringer et al., 2013; Sakai et al., 2013b). However, based on the *in vivo* experimental CDV infection of Cynomolgus macaques (*Macaca fascicularis*) in the presence of MeV immunity, macaques were partially cross protected from the CDV challenge (De Vries et al., 2014). This suggests that although CDV can readily infect primates, MeV immunity is protective and that CDV infection could be self-limiting. Transferring this result to humans, there is a potential risk of CDV infection in people who lack cross-protective MeV immunity due to non-vaccination and vaccine failures (Haralambieva et al., 2015) or due to the absence of vaccination in the possible post-eradication era (Holzmann et al., 2016).

“Emerging viruses” could reportedly arise via the cross-species transmission of viruses from animals into humans (Wolfe et al., 2007). Novel studies, both structural and bioinformatic, suggest that just a single amino acid change in a protein sequence could be enough to overcome the restriction in using cellular receptors among two different hosts, such as humans and ruminants (Abdullah et al., 2018). A unique mutation in the CDV H protein *in vitro* enables this pathogen to infect cells expressing the human SLAM receptor (Otsuki et al., 2013). Moreover, if we embrace the hypothesis that CDV evolved from MeV, it could be possible that a CDV descendant could be able to re-infect humans because of the continuous evolution of both the virus and humans, as has been previously suggested in other models even though the ancestral “jumper virus” had disappeared from earth time ago (Emerman and Malik, 2010).

Furthermore, one of the most interesting results presented by Uhl et al. is the optimization of both the CDV and MeV genes to human codon usage bias (CUB), suggesting that CDV codon usage is closer to human CUB than canine CUB because the virus or its progenitor, most likely MeV, was initially adapted to humans (Uhl et al., 2019). CUB refers to the phenomenon wherein some synonymous codons are used more often than others and how this preference varies within and among species (Behura and Severson, 2013). In RNA viruses, codon usage is under selection because the viruses are completely dependent on host tRNAs and the bias results from viruses matching the codon usage of their hosts (Jenkins and Holmes, 2003). Evolution can sometimes favor viruses that match their host codon usage to promote the replication speed and adaptation to the host as has been reported in other RNA viruses (Goni et al., 2012; Lauring et al., 2012; Di Paola et al., 2018; Freire et al., 2018).

Finally, we would like to argue that some other factors must be considered in the possible zoonotic scenario of CDV. Cross neutralization between MeV and CDV has been recognized since many years (Brown and Mccarthy, 1974), and this premise has existed for more than half a century when the MeV vaccine was used to protect pups against CDV at an age when passive maternal immunity often interfered with CDV vaccination (Baker et al., 1966; Brown et al., 1972). Nevertheless, the use of a commercial dual CDV/MeV vaccine is still recommended for vaccination in the presence of maternal immunity, and the vaccine has been useful against clinical measles disease in non-human primates (Christe et al., 2019). Hence, one may speculate that MeV herd immunity avoids CDV jump and possible readaptation to humans via transmission through dogs or wildlife animals.

## Concluding Remarks

The evolution and origin of viral pathogens cannot be easily studied; hereafter, a multidisciplinary approach is necessary to understand and perhaps predict new possible viral threats to humans. Due to their peculiar biology, viral pathogens such as CDV represent a unique model for understanding interspecies jumping and zoonotic potential of viral agents very close to the human population. Besides the traditional molecular phylogenetic studies and the paleopathology works, researchers must adopt different approaches to study CDV origin and current viral and host requirements for interspecies jumping. The introduction of computational methods, such as structural bioinformatics and paleovirology studies, could help in the prediction and prevention or at least provide a better understanding of this emerging, and perhaps, zoonotic disease from a different perspective considering not only sequencing data but also structures and functions as key information to this aim.

## Author Contributions

All authors listed have made a substantial, direct and intellectual contribution to the work, and approved it for publication.

### Conflict of Interest Statement

The authors declare that the research was conducted in the absence of any commercial or financial relationships that could be construed as a potential conflict of interest.
